# Association between recreational video gaming and auditory spatial performance in young adults: A comparative study using virtual acoustic space identification

**DOI:** 10.1371/journal.pone.0360959

**Published:** 2026-11-11

**Authors:** Prajwal Kumar Eranna, Ritik Srivastava, Kavassery Venkateswaran Nisha, Swati Goyal, Jim Saroj Winston, Prashanth P Prabhu

**Affiliations:** 1 Nitte Institute of Speech and Hearing (NISH), KS Hegde Medical Academy, Nitte (Deemed to be University), Mangalore, India; 2 Anand Hearing Care Pvt Ltd, Delhi, India; 3 Center of Hearing Sciences, COE-HS, All India Institute of Speech and Hearing (AIISH), Mysore, India; 4 Department of Audiology, All India Institute of Speech and Hearing (AIISH), Mysore, India; Towson University, UNITED STATES OF AMERICA

## Abstract

**Purpose:**

Video gaming is increasingly popular, especially among younger populations. Beyond entertainment, it has been demonstrated to offer therapeutic, medical, cognitive, educational, and health-related benefits. Modern digital-video games are incorporating advanced spatial audio features, which are crucial for locating sounds and navigating complex environments within the gaming interface. Repeated exposure to such immersive auditory environments may influence spatial hearing abilities, although the extent to which these effects extend beyond the gaming context remains unclear.

**Method:**

A cross-sectional between-group comparative design was employed with normal-hearing young adults: twenty-five gamers (≥6 hours/day of gaming for at least 6 months) and twenty-five non-gamers (< 1 hour/week). Spatial hearing was assessed using the Virtual Acoustic Spatial Identification test, in which participants identified virtual sound sources from eight azimuths using a visual interface. Outcome measures included overall and azimuth-specific accuracy, sensitivity (d′), reaction time, and spatial error patterns derived from an 8 × 8 confusion matrix. Data were analyzed using linear and general linear mixed-effect models while accounting for repeated observations within participants.

**Results:**

Across the models, residual diagnostics indicated acceptable overall model fit. Models revealed significantly higher localization accuracy and sensitivity, as well as faster reaction times, in gamers than non-gamers (*p* < .05). Significant Group × Azimuth interactions for accuracy and sensitivity indicated location-specific advantages, with gamers outperforming non-gamers at the left posterior (L135°) and left lateral (L45°) azimuths. Reaction time advantages were observed across all azimuths, whereas error analysis showed significantly fewer left-hemifield localization errors among gamers.

**Conclusion:**

The findings demonstrate an association between recreational video gaming and superior auditory spatial performance, particularly in the left hemifield. Although the underlying mechanisms remain unclear. These findings may reflect experience-related differences in auditory spatial processing and warrant further investigation using neurophysiological and intervention-based approaches.

## Introduction

Online video gaming has become one of the most popular leisure activities among young people worldwide [1], with approximately 3.32 billion people playing video games as of 2026 [2]. In India alone, a recent survey by Cyber Media Research across Tier 1, Tier 2, and Tier 3 cities [3] reported that 74% of Gen Z users (13–28 years (Generation Z refers to the demographic cohort born between 1997 and 2012)) spend at least six hours per week gaming on their phones. Beyond entertainment, research indicates that gaming offers therapeutic, medical, cognitive, educational, and health-related benefits [4,5].

While traditionally viewed as entertainment, digital games have recently evolved into powerful platforms for health-related applications, supporting cognitive training, physical rehabilitation, psychological therapy, and disease management [6]. Research demonstrates that video games can promote health outcomes by improving disease self-management, enhancing physical activity, providing distraction from discomfort, and supporting skills training for both patients and clinicians [6]. Gamified interventions have also shown promise in auditory-verbal rehabilitation, particularly for children with hearing challenges, where the engaging, interactive nature of games enhances motivation and adherence to training protocols [7,8].

A pivotal development in gaming technology is the integration of sophisticated spatial audio cues. Modern digital video games are increasingly incorporating advanced audio features into their games [9] with sophisticated spatial audio cues to enhance player immersion, requiring users to frequently localize and interpret sounds from multiple virtual directions, often under high attentional demand. Consequently, gamers are routinely exposed to complex virtual auditory environments that rely heavily on spatial cue processing. While other real-world activities may also engage auditory spatial processing, video gaming provides prolonged and repeated exposure to headphone-based virtual spatial-audio environments, making it a useful model for examining potential associations between virtual auditory experience and spatial hearing performance. However, whether such prolonged exposure is associated with differences in auditory spatial performance that extend beyond virtual listening environments remains insufficiently understood.

Sound localization in three-dimensional (3D) space involves azimuth (horizontal plane), elevation (vertical plane), and distance (depth). Localization relies on multiple acoustic cues shaped by the interaction of sound with the listener’s head, torso, and external ears. Binaural cues, including interaural time differences (ITDs) and interaural level differences (ILDs), provide important information for horizontal-plane localization, whereas spectral cues produced by the pinnae and other anatomical structures contribute particularly to elevation and front–back discrimination [10]. These spatial cues are collectively represented in the acoustic filtering captured by head-related transfer functions (HRTFs), which describe how sounds from different spatial locations are modified before reaching the listener’s ears. In gaming contexts, HRTF-based 3D audio rendering is increasingly used to simulate spatially realistic sound environments, allowing virtual sound sources to be perceived from different directions and contributing to immersive auditory experiences.

Spatial hearing skills are fundamental for navigating and interacting with the real-world auditory environment, enabling individuals to locate and identify sound sources in complex listening situations. Psychoacoustic assessments, such as the Virtual Acoustic Space Identification (VASI) test, offer a reliable and controlled means to simulate spatial listening challenges and quantify sound localization abilities [11]. While prior studies highlight the educational and rehabilitative potential of audio-based virtual environments [12,13], there is limited empirical evidence on whether regular recreational exposure to virtual spatial audio is associated with measurable differences in auditory spatial performance.

Emerging research suggests a connection between playing video games in spatially rich environments and enhanced spatial hearing. AlWhaibi et al. [14] reported that frequent gamers exhibit improved self-reported spatial abilities and greater adaptation to spatial challenges compared with infrequent gamers, particularly in action- and navigation-heavy games. The questionnaire also explored participants’ perceptions of whether gaming-related experiences translated to real-world spatial abilities, such as navigation and attention to detail. However, these findings were based on self-reported measures and do not establish whether such associations extend to objectively measured auditory spatial processing. Similarly, Parmar et al. [15] described the development of virtual reality games for spatial hearing training in children and young people with bilateral cochlear implants and the assessment framework for a planned randomized controlled trial to evaluate the effectiveness of the intervention, targeting outcomes such as spatial speech-in-noise perception, sound localization, and spatial release from masking. However, post-intervention efficacy outcomes were not reported in that article. Additional studies have highlighted that action gaming is linked to improved auditory and visual localization and that active, repeated gameplay fosters perceptual learning in sound localization tasks. Reviews of immersive virtual reality research by Gittinger & Wiesche [16] have reported improvements in certain visual-spatial abilities following interaction with virtual environments, particularly in mental rotation and perspective-taking/spatial orientation, with some studies also reporting gains in spatial visualization and distance perception. These effects were not uniform across studies, with findings indicating both advantages for high-spatial-ability learners and disadvantages for low-spatial-ability learners. At the same time, several studies suggested that interactive virtual reality may have training potential, particularly for learners with lower baseline spatial ability. However, the findings were heterogeneous and were primarily based on visual-spatial measures, and the extent to which such effects generalize across different spatial domains and persist over time remains unclear.

Despite growing interest in the cognitive and perceptual benefits of video gaming in attention [17], spatial resolution, and visual-motor coordination [18,19], evidence regarding the influence of video gaming on auditory spatial processing remains limited and fragmented. Existing studies have primarily relied on self-reported measures of spatial awareness, focused on visual-spatial abilities, or described the assessment framework for a planned RCT to evaluate the effectiveness of gaming-based interventions in clinical populations [14,15,18,19], although auditory spatial abilities were least explored. Consequently, it remains unclear whether long-term recreational exposure to spatially rich gaming environments is associated with measurable differences in auditory spatial perception among healthy individuals.

Investigating recreational gamers, therefore, provides an opportunity to examine whether repeated exposure to complex virtual auditory environments is associated with differences in virtual auditory spatial performance. Unlike structured rehabilitation or laboratory-based training programs, recreational gaming represents a naturally occurring activity that involves repeated exposure to spatialized sounds and localization demands within virtual environments. However, similar auditory spatial demands may also arise in non-virtual recreational activities, and the specific contribution of prolonged exposure to virtual, headphone-based spatial-audio environments remains unclear. The present study, therefore, focuses specifically on whether individuals familiar with virtual acoustic cues (gamers) differ from those less / unfamiliar with such cues (non-gamers) in virtual auditory spatial performance. Because both groups were assessed within the same virtual acoustic environment, the findings do not address whether any observed differences extend to non-virtual auditory spatial perception or real-world listening. Given these considerations, the present study aimed to compare auditory spatial-processing abilities between recreational gamers and non-gamers using the VASI test. In addition to conventional localization accuracy, the study examined sensitivity, reaction time, and spatial error patterns to provide a comprehensive characterization of auditory spatial performance. By determining whether individuals with extensive experience in gaming environments that rely on virtual spatial audio are associated with differences in virtual auditory spatial perception, this study may inform future research exploring the potential of gaming-inspired auditory training paradigms.

## Materials and methods

### Participants

A cross-sectional between-group comparative study design was employed to compare the performance of gamers and non-gamers in the VASI test. Power analysis was conducted using G*power software [20], based on the observed effect size (Cohen’s d = 0.94) from a parent article [21], to estimate the appropriate sample size. Using a significance level of 0.05 and a power of 80%, the required sample size was calculated to be approximately 19 participants per group, as determined by the software for two-sided independent t-tests.

A total of 50 individuals participated in the study, comprising 25 gamers (mean age = 27.04 years, SD = 1.98) and 25 non-gamers (mean age = 26.32 years, SD = 1.58). Participants were recruited through purposive sampling from local gaming communities for the gamers group, and age-matched non-gamers were recruited from university student populations. Recruitment efforts included targeted announcements on university bulletin boards, social media groups related to gaming, and direct outreach to gaming clubs and associations. Interested individuals were screened for eligibility via a structured interview designed to capture background characteristics and gaming-related habits (S1 Appendix). The screening assessed variables including the average number of hours spent gaming per day, duration of gaming at the reported frequency, primary gaming platform, use of high-quality headphones, reliance on spatial auditory cues during gameplay, and the predominant genres of games played.

To be classified as a gamer, participants were required to meet the following criteria: (a) engagement in video gaming for a minimum of six hours per day for over six months preceding the testing, (b) consistent use of high-quality headphones supporting virtual surround spatial audio features and having latency below 20ms, and (c) a primary preference for First person shooter (FPS) games (e.g., Battlegrounds Mobile India (BGMI)) or similar action/navigation-heavy titles known to contain rich spatial auditory cues. This stringent criterion was adopted to ensure the recruitment of participants with consistent exposure to gaming environments and spatial-audio cues, rather than occasional gamers. The gamers’ group had a median gaming experience of 15 months, and smartphones and laptops were the predominant gaming platforms used by participants. Participants were classified as non-gamers if they reported engaging in video gaming for less than one hour per week in the preceding six months and did not use high-quality headphones or devices equipped with spatial audio features during gameplay. Individuals in this group reported no regular engagement with FPS action, or navigation-based games that demand intensive auditory spatial processing. Their gaming activity, if any, was restricted to casual or puzzle-based games, such as mobile application games, which do not rely heavily on sound localization cues. Additionally, individuals with prior competitive or professional gaming experience, as well as those with extensive music training or professional experience in audio engineering, were excluded from both the groups to minimize potential confounding from specialized gaming or auditory experience that could independently influence spatial perception.

Furthermore, all the above-selected participants underwent audiological screening to ensure their hearing sensitivity was within normal limits, as confirmed by air- and bone-conduction pure-tone thresholds of ≤ 15 dB HL at octave frequencies from 250 Hz to 8 kHz [22]. Middle ear function was assessed through immittance evaluation, and only participants demonstrating type-A tympanograms were included in the study. Outer hair cell integrity was confirmed by the presence of transient-evoked otoacoustic emissions (TEOAEs). To further rule out auditory processing difficulties, the Screening Checklist for Auditory Processing in Adults (SCAP-A) [23] was administered, and only those who scored below 6 were considered. In addition, participants with known neurological or psychiatric disorders or a history of ototoxic drug use were not included.

Written informed consent was obtained from all participants prior to initiation of data collection, and the study procedures were reviewed and approved by the [Institutional Ethics Committee/Review Board, Approval No. SH/IRB/P.40/2025–26], ensuring compliance with the Declaration of Helsinki. Participants were recruited and the data collection was carried out between 15/09/2025 and 15/03/2026.

### Procedure

Spatial acuity of participants in both groups was assessed using the VASI test, following procedures similar to those described by Nisha & Kumar [11]. This test was chosen as it complements the localization test using real source identification [24]. Sampath et al. [24] has also demonstrated comparable spatial-acuity assessment between VASI-based virtual sound-source identification using standardized HRTFs and free-field sound-source identification in adults with normal hearing sensitivity. The test comprised of eight virtual sound-source locations, all distributed along the horizontal (azimuthal) plane: midline front (0°), midline back (180°), right 45° (R45), right 90° (R90), right 135° (R135), left 45° (L45), left 90° (L90), and left 135° (L135). These virtual stimuli were generated by multiplying the HRTF data from Sound Lab’s 3-dimensional (SLAB 3D) default database corresponding to each azimuth with a 250-ms white noise signal, thereby simulating sound locations in a virtual three-dimensional space presented over headphones. The SLAB 3D, version 6.7.3b, was implemented to generate the VASI stimuli [25]. The use of non-individualized HRTF-based stimuli is consistent with the established VASI methodology and provides a consistent stimulus configuration across participants [11–13,21,24].

A custom code running on MATLAB Online, Version R2023b [26] was used to deliver these stimuli in randomized order and to record participant responses. Each azimuth was presented 10 times at 65 dB SPL using circumaural headphones (Sennheiser HDA 300, Wedemark, Germany), resulting in a total of 80 presentations per participant, randomized across the session. Participants were instructed to select the perceived sound location by clicking the corresponding azimuth on a response window displayed in the MATLAB user interface, which automatically recorded their responses.

Before the actual VASI test, a familiarization phase was provided to acquaint them with the virtual auditory percepts and the response interface. Unlike the test phase, participants were not required to identify the source location from a presented sound. Instead, they actively explored the stimuli by clicking on different azimuth locations on the interface, upon which the corresponding virtual sound was presented. This allowed participants to freely explore and repeatedly listen to the spatial percepts associated with each azimuth until they were familiar with the sound-location correspondence. All the participants (both gamers and non-gamers) underwent this familiarization phase before proceeding to the testing phase. The familiarization phase was self-paced, and the formal test commenced once participants indicated that they were comfortable with the task, and no criterion was applied during familiarization.

### Scoring and analysis

The responses were stored in .xls format, with each file containing the target azimuths and the corresponding participant responses for further analysis. Each trial that was correctly identified was assigned a score of 1, while incorrect responses were assigned a score of 0. An 8 × 8 target-response grid, also known as a confusion matrix, was formulated using an advanced filter option in Microsoft Excel. The resulting confusion matrix was used to quantify the perceptual confusions by examining both error patterns and accuracy. Accuracy was computed as the total number of correct responses out of 10 trials for each azimuth (location-specific accuracy) and as the total number of correct responses out of all trials (80) across all azimuths (overall VASI accuracy). Reaction times were extracted for all correctly identified trials, allowing computation of both location-specific reaction times and overall reaction time for the VASI test.

In addition to raw accuracy scores, Sensitivity (d’) was also derived from the 8 × 8 target-response confusion matrix utilizing principles from signal detection theory (SDT) [27]. Although SDT was originally developed for binary detection paradigms, it has subsequently been extended to classification and identification tasks involving multiple stimulus categories. Accordingly, the present analysis employed a one-versus-all approach, in which each azimuth was treated as the target class and contrasted against all remaining azimuths. For each azimuth, the hit rate was calculated as the proportion of trials presented at that target location that were correctly identified, whereas the false alarm rate was calculated as the proportion of trials presented at all other locations that were incorrectly identified as the target location. Extreme hit and false-alarm rates (0 or 1) were corrected using a log-linear adjustment to avoid infinite d′ values [28]. Azimuth-specific sensitivity was then calculated using:


$$\begin{array}{c} d^{\prime }=Z\text{\textasciitilde{}}\left(Hit\;Rate\right)\text{\textasciitilde{}}-\text{\textasciitilde{}}Z\text{\textasciitilde{}}\left(False\;Alarm\;Rate\right) \end{array}$$

where *Z* denotes the inverse of the standard normal cumulative distribution function. This procedure provided a location-specific measure of discrimination sensitivity that is less influenced by response bias than raw localization accuracy. An overall sensitivity score was subsequently obtained by averaging the d′ values across all eight azimuths.

Additionally, error analysis was conducted to classify and quantify specific types of spatial localization errors observed in the VASI test using the 8 × 8 confusion matrix. The errors were categorized into front-back confusions, back-front confusions, right intra-hemifield errors, and left intra-hemifield errors. Front-back confusions were calculated by counting the instances where a sound originating from the 0° azimuth was mislocalized as coming from the 180° azimuth. In contrast, back-front confusions were determined by the number of 0° responses when the sound actually originated from 180°. Right intra-hemifield errors were assessed by summing mis-localizations among the right hemifield azimuths at R45°, R90°, and R135° (R90° and R135° responses for an R45° target + R90° and R45° for an R135° target + R45° and R135° for an R90° target). Similarly, left intra-hemifield errors were calculated by including confusions among L45°, L90°, and L135° azimuths (L90° and L135° responses for an L45° target + L90° and L45° for an L135° target + L45° and L135° for an L90° target). Front–back and back–front confusions were treated as distinct error categories because such reversals are common in sound localization [29] when interaural cues are limited and listeners rely more on pinna-derived spectral cues. This separation allowed front–back errors to be examined independently from intra-hemifield errors. A flowchart of the procedure used in the present study is represented in Fig 1.

**Fig 1:**
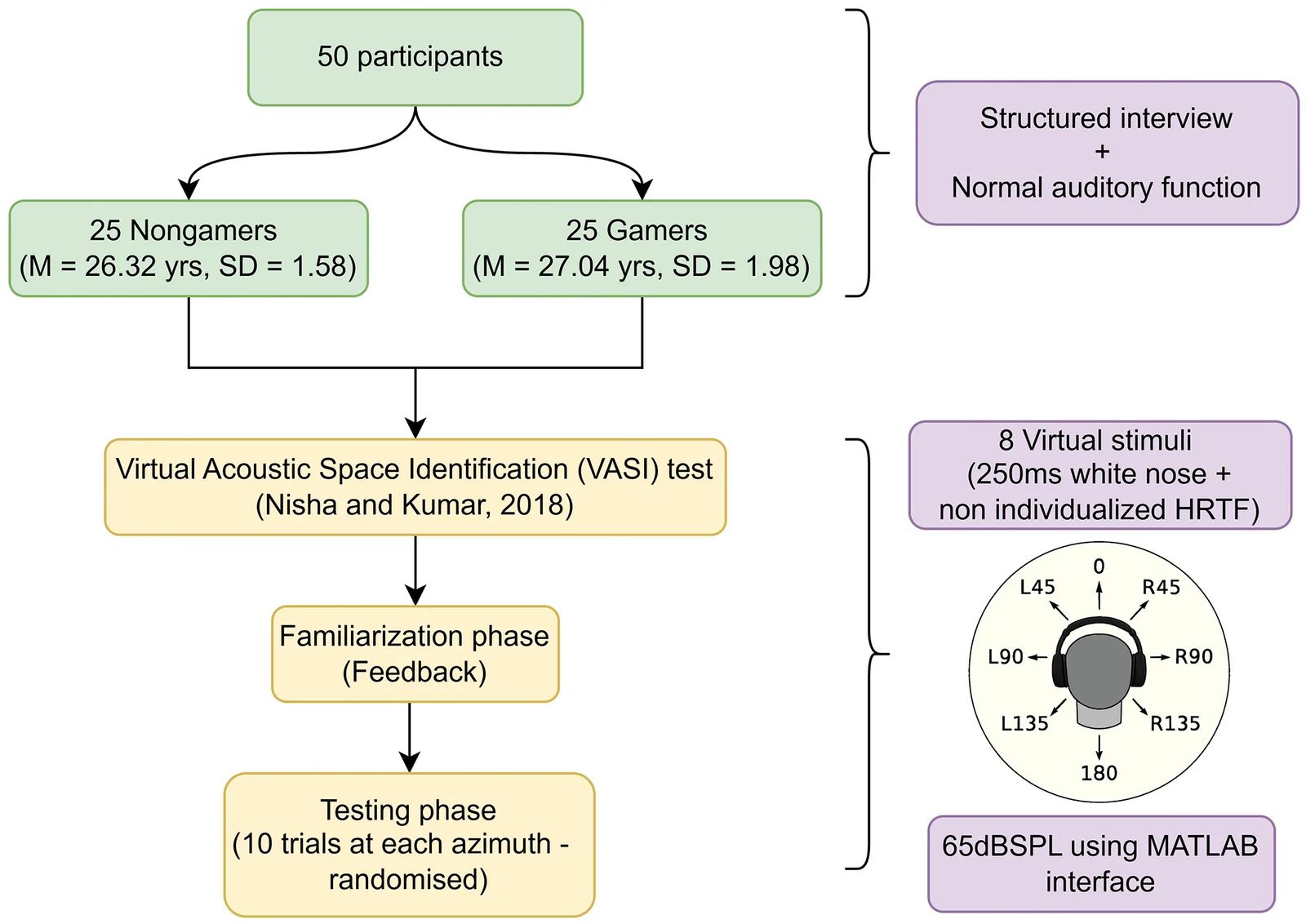
A Flowchart of the methodology used in the study.

### Statistical analysis

Statistical analyses were performed using R version 4.3.1 [30]. Linear mixed-effects regression (LMER) analyses were conducted using the lme4 package version 1.1–34 [31]. Separate mixed-effects models were fitted for localization accuracy, sensitivity, and spatial-error analysis measures to examine the effects of azimuth, error type and group, while accounting for repeated observations within participants via participant-specific random intercepts.

For localization accuracy and sensitivity, fixed effects included Azimuth, Group (gamers vs. non-gamers), and their interaction. For error analysis, fixed effects included Error Type (right hemifield, left hemifield, front-to-back confusions, and back-to-front confusions), Group, and their interaction. Models were estimated using restricted maximum likelihood (REML).

Overall participant-level performance was compared between groups using the Mann–Whitney U test, with each participant contributing a single score aggregated across all eight azimuths. In contrast, mixed-effects models examined azimuth-specific performance while accounting for repeated observations within participants and testing Group, Azimuth, and their interaction. Thus, the two approaches provided complementary assessments of overall and azimuth-specific group differences.

Reaction time data demonstrated positive skewness and violated assumptions of normality. Accordingly, reaction time was analyzed using a generalized linear mixed model (GLMM) with a Gamma distribution and a log link function, estimated via maximum likelihood with the Laplace approximation. The Gamma-family model was selected based on methodological recommendations for reaction time analysis and because it demonstrated superior residual diagnostics compared to Gaussian models, which reflect underlying sequential processing mechanisms [32–34].

Statistical significance for fixed effects was evaluated using Type III Wald chi-square tests with Satterthwaite/Kenward–Roger approximations for degrees of freedom. Estimated marginal means (EMMs) and Bonferroni-corrected post hoc pairwise comparisons were obtained using the emmeans package [35]. Effect sizes for pairwise comparisons were estimated from contrasts of model-derived estimated marginal means and standardized using the residual standard deviation from the corresponding fitted mixed-effects model, with 95% confidence intervals [36]. For reaction-time analyses, the EMMs and pairwise contrasts were obtained on the log scale because of the Gamma distribution with a log link, and the corresponding model-based residual standard deviation was used for effect-size standardization. Marginal and conditional R² values were computed to estimate the variance explained by fixed effects alone and by the combined fixed- and random-effects structure, respectively.

Model assumptions were evaluated using simulation-based residual diagnostics generated through the Diagnostics for Hierarchical Regression Models using Randomized Quantile Residuals (DHARMa) package in R [37]. Residual diagnostics included Kolmogorov–Smirnov tests for residual uniformity, dispersion tests, outlier diagnostics, and graphical inspection of residual distributions and QQ plots.

## Results

### Localization accuracy

The fitted LMER model for localization accuracy was:


$$\begin{array}{c} {}[Accuracy\text{\textasciitilde{}}∼\text{\textasciitilde{}}Azimuth*Group\text{\textasciitilde{}}+\text{\textasciitilde{}}\left(1|Participant)\right] \end{array}$$

Before examining azimuth-specific effects, total accuracy scores summed across all azimuths were compared using the Mann-Whitney U test, which showed significantly higher overall localization accuracy in gamers than non-gamers (*Z* = –2.23, *p* = .027, *r*_*e*_ = .31), indicating a moderate group difference in spatial localization performance as depicted in Fig 2.

**Fig 2:**
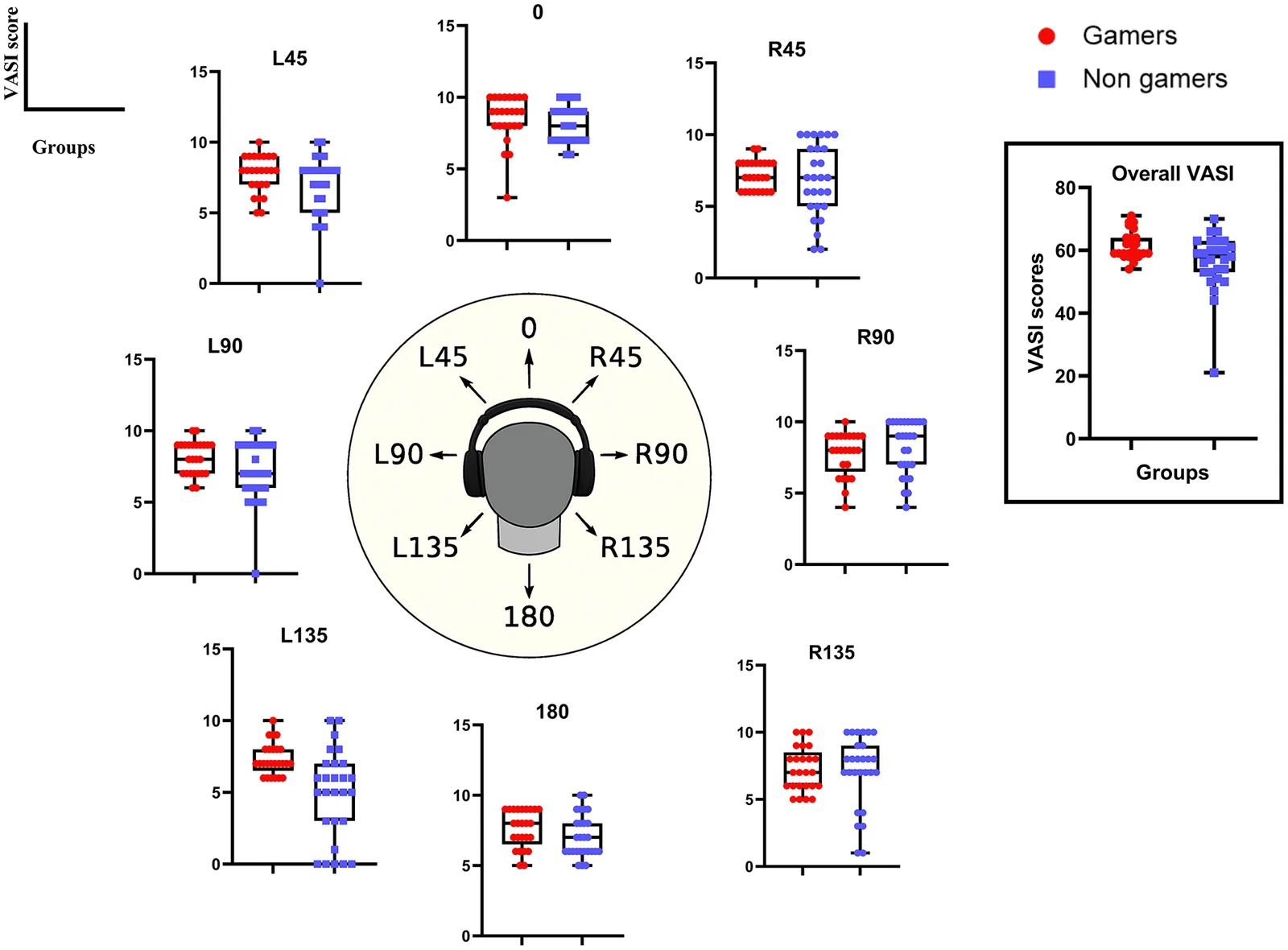
Boxplots depicting VASI accuracy scores along with individual data for each of the eight virtual sound locations evaluated in the study, with red representing gamers and blue denoting non-gamers. The central schematic illustrates the VASI test interface and spatial layout of all virtual azimuths around a dummy head. Surrounding panels display the distribution of location-specific VASI scores, while the outer panel depicts overall VASI accuracy.

Results of residual diagnostics showed that the residual uniformity (*p* = .168) and dispersion (*p* = .64) tests were non-significant. The random-effects structure demonstrated meaningful between-subject variability in localization accuracy scores. The participant-level random intercept variance was 0.47 (SD = 0.69), whereas the residual variance was 3.07 (SD = 1.75), indicating that a portion of the variability in localization accuracy was attributable to stable inter-individual differences. The model explained a moderate proportion of variance in localization accuracy, with a marginal R² of 0.16 and a conditional R² of 0.27.

The analysis revealed significant main effects of Azimuth [χ²(7) = 54.22, *p* < .001, η_p_² = 0.13, 95% CI (0.06, 0.19)], and Group [χ²(1) = 6.56, *p* = .010, η_p_² = 0.12, 95% CI (0.00, 0.29)], indicating that localization accuracy varied across spatial locations and that gamers demonstrated overall higher localization accuracy than non-gamers. Importantly, a significant Azimuth × Group interaction was observed [χ²(7) = 21.41, *p* = .003, η_p_² = 0.06, 95% CI (0.01, 0.09)], suggesting that the magnitude of group differences depended on azimuth location.

Bonferroni-corrected post hoc comparisons demonstrated that gamers exhibited significantly greater localization accuracy than non-gamers, specifically at the L135° azimuth (*p* < .001, *d* = 1.41, 95% CI [0.81, 2.00]), as shown in Table 1. Although the estimated marginal means were numerically higher for gamers at seven of the eight azimuths, with the exception of R90°, where non-gamers showed a numerically higher estimated marginal mean, these differences did not reach statistical significance following Bonferroni correction. Thus, the observed Azimuth × Group interaction reflected variation in the magnitude and direction of group differences across azimuths, with the only statistically significant between-group difference observed at L135°.

**Table 1:** Estimated marginal means (EMMs) of localization accuracy across azimuths, along with Bonferroni-corrected post hoc comparisons between gamers and non-gamers.

Azimuth	Gamers Mean (SE)	Non-gamers Mean (SE)	Mean Difference	*t* ratio	*p* value	Model-based standardized effect size	95% CI
@0°	8.52 (0.38)	8.19 (0.36)	0.34	0.64	0.52	0.19	[-0.40, 0.78]
@180°	7.60 (0.38)	7.07 (0.36)	0.53	1.01	0.32	0.30	[-0.29, 0.89]
L135°	7.32 (0.38)	4.85 (0.36)	2.47	4.73	<0.001	1.41	[0.81, 2.00]
L45°	7.72 (0.38)	6.89 (0.36)	0.83	1.59	0.11	0.47	[-0.11, 1.06]
L90°	8.12 (0.38)	7.11 (0.36)	1.01	1.93	0.05	0.58	[-0.01, 1.16]
R135°	7.24 (0.38)	7.07 (0.36)	0.17	0.32	0.75	0.09	[-0.49, 0.68]
R45°	7.20 (0.38)	6.85 (0.36)	0.35	0.67	0.51	0.20	[-0.39, 0.79]
R90°	7.76 (0.38)	8.19 (0.36)	−0.43	−0.81	0.42	−0.24	[-0.83, 0.34]

*Note* SE = standard error.

Results of residual diagnostics indicated a significant outlier test (*p* = .007). Given the potential influence of extreme observations, an additional analysis was conducted after excluding the observations identified as statistical outliers, as well as the extreme observations highlighted during data review. The results remained consistent with the primary analysis, with significant main effects of Azimuth [χ²(7) = 66.10, *p* < .001, η_p_² = 0.17, 95% CI (0.09, 0.24)] and Group [χ²(1) = 7.32, *p* = .007, η_p_² = 0.14, 95% CI (0.01, 0.33)], as well as a significant Azimuth × Group interaction [χ²(7) = 27.33, *p* < .001, η_p_² = 0.08, 95% CI (0.02, 0.13)]. Importantly, the group difference at L135° remained statistically significant (mean difference = 2.64, *p* < .0001), indicating that the observed group difference at this location was not solely attributable to the excluded extreme observations. Refer to the S1text for the complete statistical results of this additional analysis.

### Localization sensitivity

A second LMER model was fitted to examine localization sensitivity:


$$\begin{array}{c} {}[Sensitivity\text{\textasciitilde{}}∼\text{\textasciitilde{}}Azimuth*Group\text{\textasciitilde{}}+\text{\textasciitilde{}}\left(1|Participant)\right] \end{array}$$

Comparison of total sensitivity across all azimuths using the Mann-Whitney U test showed a significantly higher overall sensitivity in gamers than non-gamers (*Z* = –2.25, *p* = .026, *re* = .31), as depicted in Fig 3.

**Fig 3:**
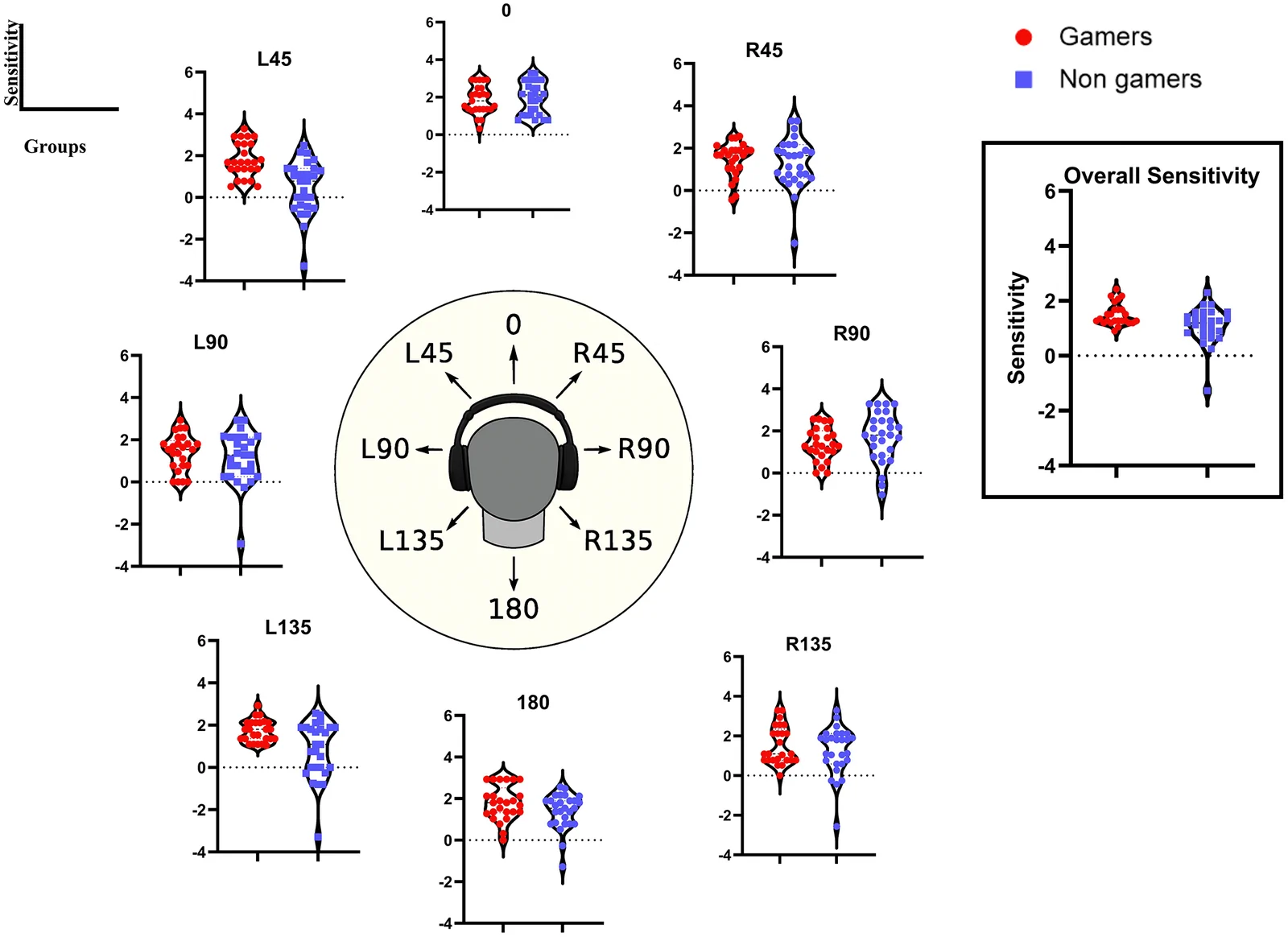
Violin plots depicting VASI sensitivity along with individual data for each of the eight virtual sound locations evaluated in the study, with red representing gamers and blue denoting non-gamers. The central schematic illustrates the VASI test interface and spatial layout of all virtual azimuths around a dummy head. Surrounding panels display the distribution of location-specific VASI sensitivity, while the outer panel depicts overall VASI sensitivity.

Findings from residual diagnostics demonstrated that the residual uniformity (*p* = .42) and dispersion tests (*p* = .72) were non-significant, suggesting the absence of systematic distributional deviations, over- or under-dispersion. The participant-level random intercept variance was 0.26 (SD = 0.51), whereas the residual variance was 0.75 (SD = 0.87). The fixed effects accounted for 13% of the variance in localization sensitivity (marginal R² = 0.13), while the full model, including both fixed and random effects, accounted for 35% of the variance (conditional R² = 0.35).

The analysis revealed significant main effects of Azimuth [χ²(7) = 27.55, *p* < .001, η_p_² = 0.07, 95% CI (0.02, 0.11)] and Group [χ²(1) = 4.70, *p* = .030, η_p_² = 0.09, 95% CI (0.00, 0.26)]. A significant Azimuth × Group interaction [χ²(7) = 34.09, *p* < .001, η_p_² = 0.09, 95% CI (0.03, 0.13)] was also observed.

Post hoc comparisons demonstrated that gamers exhibited significantly greater localization sensitivity than non-gamers, specifically at L45° [*p* < .001, *d* = 1.50, 95% CI (0.86, 2.15)] and L135° [*p* = .001, *d* = 1.06, 95% CI (0.42, 1.70)]. Both comparisons demonstrated large effect sizes, indicating substantial group differences at these spatial locations. Estimated marginal means further showed that non-gamers exhibited markedly reduced sensitivity in the left hemifield relative to gamers, as shown in Table 2.

**Table 2:** Estimated marginal means (EMMs) of localization sensitivity across azimuths, along with Bonferroni-corrected post hoc comparisons between gamers and non-gamers.

Azimuth	Gamers Mean (SE)	Non-gamers Mean (SE)	Mean Difference	*t* ratio	*p* value	Model-based standardized effect size	95% CI
@0°	1.86 (0.20)	1.96 (0.19)	−0.10	−0.35	0.73	−0.11	[-0.75, 0.52]
@180°	1.78 (0.20)	1.40 (0.19)	0.38	1.36	0.18	0.44	[-0.20, 1.07]
L135°	1.73 (0.20)	0.81 (0.19)	0.92	3.29	0.001	1.06	[0.42, 1.70]
L45°	1.81 (0.20)	0.51 (0.19)	1.30	4.67	<.001	1.50	[0.86, 2.15]
L90°	1.40 (0.20)	1.13 (0.19)	0.27	0.98	0.33	0.32	[-0.32, 0.95]
R135°	1.53 (0.20)	1.26 (0.19)	0.27	0.99	0.33	0.32	[-0.32, 0.95]
R45°	1.48 (0.20)	1.32 (0.19)	0.15	0.56	0.58	0.18	[-0.46, 0.81]
R90°	1.38 (0.20)	1.73 (0.19)	−0.35	−1.25	0.21	−0.40	[-1.04, 0.23]

*Note* SE = standard error.

### Reaction time

Reaction time was analyzed using a Gamma GLMM with log link function:


$$\begin{array}{c} {}[Reaction\text{\textasciitilde{}}time\text{\textasciitilde{}}∼\text{\textasciitilde{}}Azimuth*Group+(1|Participant)] \end{array}$$

Residual dispersion testing was non-significant (*p* = .56), indicating the absence of over- or under-dispersion within the model. Similarly, the outlier test was non-significant (*p* = .786), suggesting that influential observations were not disproportionately affecting model estimates. Although the residual uniformity reached statistical significance (*p* = .031), inspection of residual plots revealed only minor deviations from ideal behavior, and the overall diagnostic profile supported an acceptable model fit for the Gamma GLMM. The participant-level random-intercept variance was 0.054 (SD = 0.23). The model explained a substantial proportion of variance in reaction time, with a marginal R² of 0.298 and a conditional R² of 0.726.

The analysis revealed significant main effects of Azimuth, [χ²(8) = 34.27, *p* < .001], and Group [χ²(1) = 28.80, *p* < .001], indicating that reaction times differed across spatial locations and that gamers demonstrated overall faster localization responses than non-gamers. However, the Azimuth × Group interaction was not statistically significant [χ²(8) = 10.74, *p* = .217], suggesting that the gamer advantage in reaction time remained relatively consistent across azimuths.

Fixed-effect estimates further demonstrated that gamers exhibited significantly lower reaction times overall [β = −0.19, SE = 0.03, *z* = −5.37, *p* < .001, 95% CI (−0.25, −0.12)]. Bonferroni-corrected post hoc comparisons revealed that gamers responded significantly faster than non-gamers at all azimuth locations (all *p* ≤ .0011). Estimated marginal means demonstrated consistently lower reaction times in gamers across all spatial locations, supporting a generalized reaction-time advantage rather than one restricted to specific azimuths, as reflected in Table 3.

**Table 3:** Estimated marginal means (EMMs) of localization reaction time across azimuths, along with Bonferroni-corrected post hoc comparisons between gamers and non-gamers.

Azimuth	Gamers Mean (SE)	Non-gamers Mean (SE)	Log Mean Difference	z ratio	p value	Model-based standardized effect size	95% CI
@0°	0.98 (0.06)	1.42 (0.06)	−0.439	−5.14	<.001	−2.33	[-3.24, -1.43]
@180°	1.05 (0.06)	1.37 (0.06)	−0.326	−3.82	<.001	−1.73	[-2.63, -0.84]
L135°	1.00 (0.06)	1.38 (0.06)	−0.380	−4.45	<.001	−2.02	[-2.92, -1.12]
L45°	0.98 (0.06)	1.36 (0.06)	−0.389	−4.56	<.001	−2.07	[-2.97, -1.17]
L90°	0.91 (0.06)	1.19 (0.06)	−0.279	−3.26	.001	−1.48	[-2.37, -0.59]
R135°	1.08 (0.06)	1.40 (0.06)	−0.321	−3.76	<.001	−1.70	[-2.60, -0.81]
R45°	0.91 (0.06)	1.39 (0.06)	−0.480	−5.63	<.001	−2.55	[-3.46, -1.65]
R90°	0.94 (0.06)	1.29 (0.06)	−0.350	−4.10	<.001	−1.86	[-2.76, -0.96]

### Spatial error pattern

A separate LMER model was fitted to examine the effects of Error Type and Group on spatial localization errors:


$$\begin{array}{c} {}[Total\text{\textasciitilde{}}Number\text{\textasciitilde{}}of\text{\textasciitilde{}}errors\text{\textasciitilde{}}∼\text{\textasciitilde{}}Errortype*Group+(1|Participant)] \end{array}$$

Residual diagnostics indicated acceptable model fit with non-significant residual uniformity (*p* = 0.14) and dispersion (p = 0.69), indicating no evidence of systematic deviation from the expected residual distribution and absence of over or underdispersion within the model. The outlier test was also non-significant (*p* = .231), indicating that influential observations were not disproportionately affecting model estimates. The participant-level random-intercept variance was 0.27 (SD = 0.52), whereas the residual variance was 6.12 (SD = 2.47). The model demonstrated a good fit to the data with a marginal R^2^ of 0.56 and a conditional R^2^ of 0.58.

The analysis revealed a significant main effect of Error Type [χ²(3) = 92.90, *p* < .001, η_p_² = 0.63, 95% CI (0.56, 1.00)], indicating that the frequency of localization errors differed substantially across error categories. Although the main effect of Group was not statistically significant [χ²(1) = 0.31, *p* = .579], a significant Error Type × Group interaction was observed [χ²(3) = 10.31, *p* = .016, η_p_² = 0.06, 95% CI (0.01, 1.00)], indicating that group differences varied across error categories.

Bonferroni-corrected post hoc comparisons demonstrated that non-gamers exhibited significantly greater left hemifield localization errors than gamers [*p* < .001, *d* = 1.10, 95% CI (0.54, 1.66)]. In contrast, group differences for back-to-front confusions, front-to-back confusions, and right hemifield errors were not statistically significant following correction for multiple comparisons. Estimated marginal means further demonstrated that left hemifield errors were significantly higher in both groups, although the magnitude of these errors was markedly greater in non-gamers, as reflected in Table 4 and Fig 4. Within-group comparisons revealed that both groups demonstrated higher left- and right-hemifield errors relative to front–back and back–front confusions, with the increase in left hemifield errors being particularly pronounced among non-gamers.

**Table 4:** Estimated marginal means (EMMs) of localization error scores across error categories, along with Bonferroni-corrected post hoc comparisons between groups.

Error Type	Gamers Mean (SE)	Non-gamers Mean (SE)	Mean Difference (Gamers – Non-gamers)	t ratio	p value	Model-based standardized effect size	95% CI
Back–Front confusion	1.80 (0.51)	1.41 (0.49)	0.39	0.56	.580	0.16	[-0.40, 0.72]
Front–Back confusion	0.88 (0.51)	1.82 (0.49)	−0.94	−1.34	.183	−0.38	[-0.94, 0.18]
Left Hemifield Error	5.92 (0.51)	8.63 (0.49)	−2.71	−3.86	<.001	−1.10	[-1.66, -0.54]
Right Hemifield Error	6.20 (0.51)	7.15 (0.49)	−0.95	−1.36	.177	−0.39	[-0.94, 0.18]

**Fig 4:**
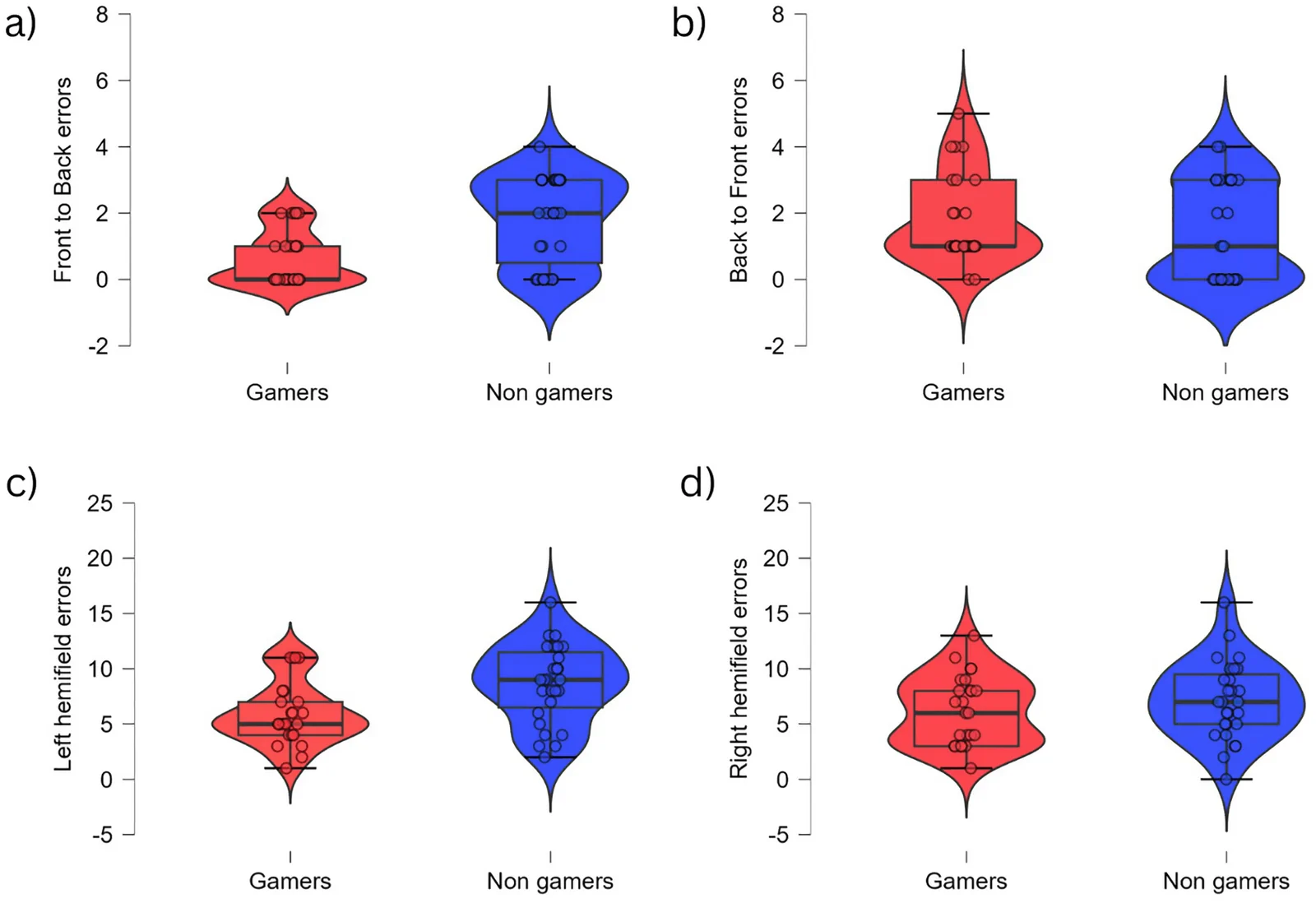
Violin plots illustrating spatial localization errors across different error types between Gamers and Non-gamers. (a) Front to Back errors, (b) Back to Front errors, (c) Left hemifield errors, and (d) Right hemifield errors. Red plots represent Gamers and blue plots represent Non-gamers. Each plot displays the distribution, median, interquartile range, and individual participant data points for each group and error type.

## Discussion

The present study aimed to investigate whether frequent engagement in video gaming, particularly within virtual environments that are rich in spatial auditory cues, influences virtual auditory spatial hearing performance as measured by the VASI test. Thus, spatial hearing performance was compared between gamers and non-gamers across multiple dimensions, including localization accuracy, sensitivity, reaction time, and spatial error patterns. Previous research has reported differences in visual-spatial performance among gamers [18], as well as multisensory enhancements [38], and training-induced changes in auditory spatial processing [13]. However, the present findings provide more specific evidence of group differences in virtual auditory spatial performance.

Gamers demonstrated significantly better overall sensitivity (Fig 3) and accuracy (Fig 2), particularly at partially lateral left-hemifield azimuths (L45° and L135°), suggesting that group differences were spatially selective rather than uniform across all spatial locations. One possible explanation is that repeated exposure to virtual auditory environments could contribute to improved processing of lateral spatial cues commonly encountered in contemporary game audio design [39]. However, the present study did not quantify the spatial distribution of game audio, and therefore, this interpretation remains speculative. Further, superior performance was observed only on the left side. Although previous studies have suggested that the right hemisphere plays an important role in auditory spatial attention and processing of contralateral auditory space [40,41], the present findings do not provide direct evidence of hemispheric specialization or neural adaptation, and this possibility warrants investigation using neurophysiological approaches. Therefore, this interpretation should be regarded as speculative, and alternative explanations, including task characteristics, HRTF asymmetries, or attentional bias, cannot be excluded.

Error analysis further supported the spatially selective nature of the findings, with gamers exhibiting significantly fewer left-hemifield localization errors than non-gamers (Fig 4). This behavioral advantage may reflect greater familiarity with interpreting spatial cues encountered in virtual listening environments rather than a specific neural mechanism.

Moreover, a reduction in front–back confusions in gamers can be explained by the inherent difficulty in localizing front sounds due to limited ITDs and ILDs, forcing reliance on ambiguous spectral cues. When faced with a challenging condition (such as a lack of ITD, ILD, or ambiguous spectral cues), gamers utilize their greater familiarity with interpreting the spectral and spatial cues available in the virtual auditory environment to identify the virtual location; however, non-gamers tend to fall short in this endeavor. In contrast, sounds arriving from behind the listeners benefit from more explicit high-frequency spectral cues shaped by the pinna, resulting in no difference in back–front confusions between the groups. Familiarity in virtual auditory spaces likely improved gamers’ interpretation of these spectral cues, enabling better resolution of front–back ambiguity.

Furthermore, reaction time analysis in the current study revealed that gamers responded significantly faster than non-gamers across all azimuths and overall. One possible explanation for this faster reaction time, coupled with greater accuracy and sensitivity in gamers, is that regular engagement with virtual auditory environments may lead to more efficient use of spatial cues and quicker localization decisions. However, this interpretation should be considered within the context of the testing environment. This observation is consistent with previous study by Nisha & Kumar [11] reporting faster response times following training in virtual auditory environments and with broader evidence linking video game experience to improved perceptual and motor performance in spatially demanding tasks [42–45]. However, because the present study assessed only behavioral outcomes, the underlying neural mechanisms cannot be determined from the current data.

The pattern of results observed in the present study aligns with existing evidence on spatial cognition and perceptual training. For instance, Green & Bavelier [18] demonstrated that habitual action video game players possess superior visual selective attention in controlled computer-based visual tasks and further showed improvement following action-game training. Their findings suggest that action-game experience can modify attentional processing beyond the specific visual features encountered during gameplay; however, whether such effects generalize to real-world three-dimensional environments or transfer across sensory modalities remains unestablished. Donohue et al. [38] further extended these findings, showing improved multisensory temporal processing among gamers, as reflected in their greater ability to discriminate the temporal synchrony and order of simple auditory and visual stimuli. Such enhanced sensitivity to fine temporal relationships across sensory modalities may be relevant to the rapid integration of auditory and visual cues during spatial perception. The observed group differences in virtual spatial discrimination may reflect more efficient perceptual or cognitive processing; however, no neural measures were obtained in the present study.

In contrast, studies focusing specifically on auditory processing have yielded mixed results. Stewart et al. [46], for example, found no significant differences between gamers and non-gamers in general auditory tasks, such as speech perception in noise. However, these tasks did not specifically require spatial separation or auditory spatial processing, limiting their direct relevance to auditory localization. More closely related to the present study are paradigms involving spatial release from masking (SRM), in which spatial separation between a target and competing sound sources facilitates target identification through the use of spatial cues for auditory scene analysis. Such tasks provide a complementary measure of spatial hearing because they require listeners to segregate competing auditory streams without requiring an explicit localization response [47–49]. Thus, comparisons between gaming experience and performance on spatially separated speech or SRM tasks may provide a more appropriate basis for evaluating whether gaming-related auditory benefits extend to spatial hearing.

Additional support for the spatially selective pattern observed in the present study comes from non-gaming localization research in the broader context of experience-dependent refinement of auditory spatial processing. Zheng et al. [50] demonstrated that localization strategies transition toward more fine-grained spatial identification with increased bilateral hearing experience in children with bilateral cochlear implants. Although their population and auditory experience differed substantially from those of the present study, their findings support the broader principle that experience can influence the precision with which acoustic space is represented. Further findings of the present study are also supported by self-reported spatial benefits, as documented by AlWhaibi et al. [14] among frequent video game players, particularly in adapting to spatial challenges and maintaining awareness of spatial elements. Complementing these subjective reports, Jordan and Dhamala [51] found greater dorsal attention network (DAN) connectivity with the salience network in gamers during sensorimotor decision-making. As the DAN supports attention to task-relevant information, enhanced engagement of this network may contribute to more efficient processing of spatial cues. Although neither study directly examined auditory localization, these findings provide a possible cognitive and neural context for the spatial differences observed between gamers and non-gamers in the present study.

Despite these converging lines of evidence, it is essential to note inconsistencies within the field. Hazzaa et al. [52] reported adverse effects of prolonged gaming on central auditory processing in children, highlighting the potential for context- and task-specific outcomes. Such discrepancies likely reflect the complex interplay among the type of game, its audio design, the cognitive demands imposed, and the player’s own attentional strategies. The findings of the current study indicate that recreational gamers demonstrate superior virtual auditory spatial performance under the present testing conditions. Whether these differences arise from gaming experience, pre-existing individual characteristics, or familiarity with virtual auditory environments remains to be determined through longitudinal and experimental studies. An important consideration when interpreting the present findings is that the gamer group had extensive experience with headphone-based virtual spatial audio, whereas the non-gamer group had minimal exposure to such listening environments. Because the VASI test also presents spatialized sounds using non-individualized HRTFs over headphones, the observed group differences may partly reflect greater familiarity with virtualized auditory cues rather than gaming experience alone. Long-term exposure to virtual acoustic environments may facilitate more efficient interpretation of HRTF-based localization cues, resulting in better performance under similar testing conditions. Therefore, the present findings should be interpreted as demonstrating an association between recreational gaming experience and auditory spatial performance within a virtual listening environment, rather than evidence that gaming itself directly improves real-world spatial hearing.

The current study employed a controlled psychoacoustic assessment, VASI, to comprehensively evaluate multiple dimensions of auditory spatial processing, including localization accuracy, sensitivity, reaction time, and error patterns. Furthermore, the use of mixed-effects modelling allowed for appropriate accounting of participant-level variability while maximizing the use of repeated-measures data across spatial locations.

Nevertheless, certain limitations should be considered when interpreting the findings. The cross-sectional nature of the study precludes causal conclusions about the effects of gaming experience, and it remains possible that individuals with superior baseline spatial abilities are more likely to engage in gaming activities, and pre-existing differences in cognitive abilities (attention) contributed to the observed group differences, warranting longitudinal or intervention-based studies. In addition, pre-existing differences in cognitive functions, such as attention, processing speed, working memory, executive functioning, and visuospatial abilities, were not directly assessed and may have contributed to the observed group differences. Consequently, the observed group differences should be interpreted as associations rather than evidence that gaming causes superior auditory spatial performance. Although gaming exposure was characterized in terms of duration, platform, game genre, and headphone use, more detailed measures of gaming history, such as cumulative lifetime experience and competitive gaming status, were not quantified. Furthermore, differences in the specific spatial-audio implementations and cues used across individual games were not standardized or quantitatively characterized. The use of standardized, non-individualized HRTFs may also have contributed to differences in familiarity with virtual spatial-audio rendering between groups, although this approach is consistent with established VASI methodology. Although individuals with extensive music training or professional audio experience were excluded from both groups to minimize potential confounding, this may limit the generalizability of the findings and prevent direct comparison of gaming-related spatial-audio experience with other forms of auditory or spatial-audio training.

Notably, the mixed-effects analyses revealed considerable individual variability, suggesting that auditory spatial performance is influenced by factors beyond gaming experience alone. Future studies should compare virtual gamers with non-virtual gamers, alongside non-gamers, using both virtual (headphone-based) and free-field localization paradigms, or individualized HRTFs, to better isolate the potential contribution of virtual spatial-audio exposure and determine whether observed differences generalize beyond virtual listening environments. Finally, as the VASI primarily assesses horizontal-plane localization, future research should examine whether the observed advantages extend to elevation perception and more complex three-dimensional listening environments.

## Conclusion

The present study demonstrates superior auditory spatial performance among gamers compared with non-gamers on the VASI test as evidenced in overall accuracy, sensitivity, and reaction time, with spatially selective differences most prominent in the left hemifield. These group differences may be related to repeated exposure to spatially rich virtual auditory environments, specifically lateral cues during gaming. However, alternative explanations, including pre-existing differences between groups and long-term familiarity with virtual auditory environments in gamers, cannot be excluded. Error analyses reveal that gamers make fewer left intra-hemifield and front-back confusions, indicating more refined spectral cue processing. Faster response times further suggest superior perceptual and cognitive processing speed in gamers. Together, findings are consistent with experience-dependent differences in auditory spatial processing but do not establish neural plasticity or causal effects of gaming. This has potential implications for leveraging gaming-based auditory training paradigms to improve rehabilitation and auditory skills. Future research should explore the influence of exposure using longitudinal designs, the effects of different game genres, and the transferability of training to real-world listening conditions to implement gaming as an intervention strategy.

## Supporting information

S1 Appendix Participant screening questionnaire used for recruitment and classification of gamers and non-gamers. (PDF)

S1 Text Additional analysis of localization accuracy following exclusion of statistical outliers and extreme observations. (PDF)
