# Supplementary material for: Association between recreational video gaming and auditory spatial performance in young adults: A comparative study using virtual acoustic space identification
Source: PLoS One. 2026 Nov 11;21(11):e0360959. doi: 10.1371/journal.pone.0360959 (PMC13754160; doi:10.1371/journal.pone.0360959)
Supplement: Supplementary file 1 [file pone.0360959.s001.pdf]

Appendix I

Participant Screening Questionnaire

Participant ID: ___________
Date of Assessment: ___________

Section A: Gaming Engagement

1. **Do you currently play video games?**
   ☐ Yes
   ☐ No
2. **On average, how many hours per day do you spend playing video games?**
   ☐ Less than 1 hour per week
   ☐ 1–2 hours per day
   ☐ 3–5 hours per day
   ☐ 6 or more hours per day
3. **For how long have you been gaming at this frequency?**
   ☐ Less than 3 months
   ☐ 3–6 months
   ☐ More than 6 months

Section B: Audio Hardware and Spatial Audio Features

4. **What type of device do you primarily use for gaming?**
   ☐ Smartphone
   ☐ Tablet
   ☐ PC/Laptop
   ☐ Gaming console (e.g., PlayStation, Xbox)
   ☐ Other (please specify): ___________
5. **Do you use high-quality headphones while gaming?**
   ☐ Yes
   ☐ No

Section C: Game Genre and Content

8. **What type(s) of games do you play most frequently?** *(Select all that apply)*
   ☐ First-Person Shooter (FPS) (e.g., BGMI, Call of Duty, Counter-Strike)

☐ Action/Adventure (e.g., Assassin's Creed, Grand Theft Auto)
☐ Battle Royale (e.g., Free Fire, Apex Legends)
☐ Role-Playing Games (RPG) (e.g., The Witcher, Final Fantasy)
☐ Strategy/Real-Time Strategy (e.g., Age of Empires, StarCraft)
☐ Puzzle/Casual games (e.g., Candy Crush, Sudoku)
☐ Sports/Racing (e.g., FIFA, Need for Speed)
☐ Other (please specify): ___________

9. **Do the games you play require you to locate sounds or rely on auditory cues to navigate or compete effectively?**
☐ Yes, frequently
☐ Sometimes
☐ Rarely
☐ No

Section D: Exclusion Criteria

10. **Have you received formal training in music (e.g., vocal or instrumental training)?**
☐ Yes
☐ No

Classification Criteria

Gamer Group:

- Engagement in video gaming for ≥6 hours per day for >6 months
- Consistent use of high-quality headphones with spatial audio features
- Primary preference for FPS or action/navigation-heavy games with rich spatial auditory cues

Non-Gamer Group:

- Gaming activity <6 hours per week in the preceding 6 months
- No use of high-quality headphones or spatial audio devices
- No regular engagement with FPS, action, or navigation-based games
- Gaming limited to casual/puzzle games with minimal auditory spatial demands

Exclusions:

- Formal music training
