# Supplementary material for: Association between recreational video gaming and auditory spatial performance in young adults: A comparative study using virtual acoustic space identification
Source: PLoS One. 2026 Nov 11;21(11):e0360959. doi: 10.1371/journal.pone.0360959 (PMC13754160; doi:10.1371/journal.pone.0360959)
Supplement: Supplementary file 2 [file pone.0360959.s002.pdf]

Supplementary Results S1. Sensitivity Analysis Following Exclusion of Outliers and Extreme Observations

To evaluate the robustness of the localization accuracy findings, the primary linear mixed-effects analysis was repeated after excluding observations identified as statistical outliers, as well as the extreme observations highlighted during data review. The same model structure was retained:

$$Accuracy \sim Azimuth \times Group + (1 \mid Participant)$$

The reanalysis yielded results broadly consistent with the primary analysis.

Table S1. Linear mixed-effects model after exclusion of outliers and extreme observations

**Effect**	**χ^2(df)**	**p**	**Partial η^2 (95% CI)**
Azimuth	39.65 (7)	< .001	0.12 [0.04, 0.18]
Group	5.49 (1)	.019	0.12 [0.00, 0.32]
Azimuth × Group	14.21 (7)	.048	0.05 [0.00, 0.08]

Model fit:

- Marginal R^2 = 0.143
- Conditional R^2 = 0.194

Table S2. Estimated marginal means and between-group comparisons

**Azimuth**	**Gamers Mean (SE)**	**Non-gamers Mean (SE)**	**Mean Difference**	**p**	**Model-based standardized effect size**	**95% CI**
@0°	8.75 (0.34)	8.22 (0.35)	0.53	.279	0.32	−0.26, 0.90
@180°	7.58 (0.34)	7.30 (0.35)	0.28	.571	0.17	−0.42, 0.75
**L135°**	**7.29 (0.34)**	**4.65 (0.35)**	**2.64**	**< .001**	**1.59**	**0.997, 2.186**
L45°	7.71 (0.34)	7.09 (0.35)	0.62	.207	0.37	−0.21, 0.96
L90°	8.17 (0.34)	7.43 (0.35)	0.73	.137	0.44	−0.14, 1.03
R135°	7.33 (0.34)	7.65 (0.35)	−0.32	.517	−0.19	−0.78, 0.39
R45°	7.12 (0.34)	7.04 (0.35)	0.08	.868	0.05	−0.53, 0.63
R90°	7.83 (0.34)	8.26 (0.35)	−0.43	.385	−0.26	−0.84, 0.33

Following exclusion of statistical outliers together with the extreme observations highlighted during data review, the overall pattern of results remained consistent with the primary analysis. Significant main effects of Azimuth and Group, as well as a significant Azimuth × Group interaction, were retained. The only statistically significant between-group difference continued to occur at the L135° azimuth, where gamers demonstrated significantly higher

localization accuracy than non-gamers (mean difference = 2.64, $p < .001$). Although effect sizes and model fit statistics changed modestly following removal of these observations, the principal conclusions of the study remained unchanged, indicating that the reported findings were robust and not driven solely by extreme observations.
